# Ethnic Minority Status, Age-at-Immigration and Psychosis Risk in Rural Environments: Evidence From the SEPEA Study

**DOI:** 10.1093/schbul/sbx010

**Published:** 2017-05-17

**Authors:** James B Kirkbride, Yasir Hameed, Konstantinos Ioannidis, Gayatri Ankireddypalli, Carolyn M Crane, Mukhtar Nasir, Nikolett Kabacs, Antonio Metastasio, Oliver Jenkins, Ashkan Espandian, Styliani Spyridi, Danica Ralevic, Suneetha Siddabattuni, Ben Walden, Adewale Adeoye, Jesus Perez, Peter B Jones

**Affiliations:** 1 PsyLife Group, Division of Psychiatry, University College London, London, UK; 2 Department of Psychiatry, University of Cambridge, Cambridge, UK; 3 Norfolk & Suffolk Foundation Trust, Norwich, UK; 4 Cambridgeshire & Peterborough Foundation Trust and NIHR Collaboration for Leadership in Applied Health Research and Care (CLAHRC) East of England, Cambridge, UK; 5 North Essex Partnership NHS Foundation Trust, Chelmsford, UK

**Keywords:** epidemiology, ethnicity, migration, urbanicity, incidence, early intervention, social determinants

## Abstract

**Objective:**

Several ethnic minority groups experience elevated rates of first-episode psychosis (FEP), but most studies have been conducted in urban settings. We investigated whether incidence varied by ethnicity, generation status, and age-at-immigration in a diverse, mixed rural, and urban setting.

**Method:**

We identified 687 people, 16–35 years, with an ICD-10 diagnosis of FEP, presenting to Early Intervention Psychosis services in the East of England over 2 million person-years. We used multilevel Poisson regression to examine incidence variation by ethnicity, rural–urban setting, generation status, and age-at-immigration, adjusting for several confounders including age, sex, socioeconomic status, population density, and deprivation.

**Results:**

People of black African (incidence rate ratio: 4.06; 95% confidence interval [CI]: 2.63–6.25), black Caribbean (4.63; 95% CI: 2.38–8.98) and Pakistani (2.31; 95% CI: 1.35–3.94) origins were at greatest FEP risk relative to the white British population, after multivariable adjustment. Non-British white migrants were not at increased FEP risk (1.00; 95% CI: 0.77–1.32). These patterns were independently present in rural and urban settings. For first-generation migrants, migration during childhood conferred greatest risk of psychotic disorders (2.20; 95% CI: 1.33–3.62).

**Conclusions:**

Elevated psychosis risk in several visible minority groups could not be explained by differences in postmigratory socioeconomic disadvantage. These patterns were observed across rural and urban areas of our catchment, suggesting that elevated psychosis risk for some ethnic minority groups is not a result of selection processes influencing rural–urban living. Timing of exposure to migration during childhood, an important social and neurodevelopmental window, may also elevate risk.

## Introduction

Some migrants and their descendants experience increased risk of schizophrenia and other psychotic disorders compared with the majority population in a given setting.^1,2^ Precise risk correlates with visible minority status, meaning that black Caribbean and African migrants and their descendants in Europe and North America experience greatest incidence.^3–6^ Rates are also 2–4 times higher in people of Pakistani and Bangladeshi origin in the United Kingdom,^7^ and Moroccans in the Netherlands.^8^ Psychosis risk may also be raised among white migrants, although research from Scandinavia suggests “European” migrants may only have up to double the risk of native-born groups.^9,10^ Nonetheless, the ethnicity of these migrants was unknown; studies of white ethnic minorities in the United Kingdom suggest risk of first-episode psychosis (FEP) may be 50% higher than in the white British population.^7,11^ No epidemiological study has, however, been conducted since substantial immigration from Eastern Europe following EU expansion in 2004, while little is known about FEP risk in other potentially marginalized groups, including minorities of Arabic origin.

Elucidation of the determinants of this variation in risk should be a mental health research priority, given that up to one-fifth of FEP worldwide may be preventable,^12^ if we could remove the causes of excess risk experienced by black and minority ethnic (BME) groups. Elevated risk is unlikely to be explained by selective migration^13,14^ or higher rates in countries of origin,^15–18^ arguing against solely genetic explanations of causation. Further, rates appear elevated to similar extents among first- and later-generation BME groups,^1^ suggesting that exposure to psychosocial adversities at all stages of the migration process (before, during, and after) contribute to underlying etiology.

To date, most epidemiological studies on FEP risk in different ethnic groups have been conducted in predominantly urban settings or national databases; studies in rural populations have generally been too ethnically homogeneous to investigate this issue. BME groups in rural areas may differ from their urban counterparts in important ways, including differential exposure to socioeconomic status (SES) and other aspects of social disadvantage, such as deprivation. Nonetheless, BME groups in rural areas may also face increased exposure to other social stressors, such as visible minority position or social isolation, but how rural living affects psychosis incidence in BME groups has received little attention to date. It is also unclear whether risk varies by generation status (first vs later-generation BME groups) in more rural populations, or, more generally, whether timing of exposure to migration among first-generation groups also affects psychosis risk. To date only 3 peer-reviewed studies have investigated this latter issue^6,9,19^; 2 found migration during childhood conferred strongest psychosis risk,^6,19^ consistent with other evidence that this may be a vulnerable window of exposure to psychosocial adversities affecting typical neurodevelopment.^20,21^ However, a third study, predominantly among European migrants to Denmark, did not.^9^

To investigate these issues, we used epidemiological data from a large, naturalistic cohort study in a rural setting in the East of England,^22^ where 20% of the population at-risk were from a BME background, half of whom were of non-British white (mostly European) origins, following a decade of EU expansion. We tested the following hypotheses:

1. Incidence would be elevated among most BME groups compared with the white British population at-risk, after adjustment for SES, population density and multiple deprivation. The magnitude of excess risk would broadly correlate with visible minority status
2. Patterns of risk would be similar in both rural and urban settings within the catchment area
3. Rates would be elevated in first- and later-generation groups to a similar extent
4. For first generation migrants, rates would be most-strongly associated with immigration in childhood and adolescence

## Methods

### Design and Setting

The Social Epidemiology of Psychoses in East Anglia [SEPEA] study prospectively identified all people, aged 16–35 years old, presenting to 6 early intervention in psychosis (EIP) services with a suspected FEP in the East of England, over a 3.5-year period. Case ascertainment was from August 1, 2009 to January 31, 2013 in the catchment area of the Cambridgeshire and Peterborough NHS Foundation Trust, and from September 28, 2009 to March 28, 2013 in the Norfolk and Suffolk NHS Foundation Trust. The region had a population of 2.4 m people in 2011, and is varied in terms of ethnicity, socioeconomic deprivation, and population density.^22^

### Case Ascertainment

EIP services were the sole referral point for anyone with suspected psychosis in the catchment area, and worked closely with a comprehensive range of sources, including primary and secondary care providers, as well as tertiary services, schools, hostels, and self-referrals. FEP cases referred to other mental health services would normally be re-directed to EIP services. We identified and followed all participants accepted into EIP care until receipt of 3 years of care, or discharge from services (for any reason), if earlier. Inclusion criteria at first referral were:

1. 16–35 years old (17–35 years in Cambridgeshire and Peterborough)
2. Resident in the catchment area, including those of no fixed abode
3. Absence of moderate or severe learning disabilities, or an organic basis to disorder
4. No previous contact with health services for psychosis, or previous treatment with anti-psychotic medication for greater than 6 months

### Diagnostic Outcomes

We initially included participants who received a clinical diagnosis according to the tenth revision of the International Classification of Diseases (ICD-10) at 6 months and 3 years into their EIP care, or at discharge from services, if sooner. These diagnoses were validated by an ethnically diverse panel of trained diagnosticians using the OPCRIT assessment,^23^ which uses a standardized, structured 90-item symptom checklist to ascertain valid and reliable research-based diagnoses from case note review.^23,24^ Participants without an OPCRIT-confirmed ICD-10 psychotic disorder (F10-33) at either time point were excluded. We classified participants according to their final OPCRIT-confirmed diagnosis as follows: all clinically relevant psychotic disorders (F10–33), nonaffective psychoses (F20–29), schizophrenia (F20), and affective psychoses (F30–33). We did not include substance-induced psychoses as a separate outcome in this article, given the small sample size, as previously reported (*N* = 30; 4.4%).^22^

### Population At-Risk

We estimated the population at-risk from the Office for National Statistics (ONS) 2011 Census of Great Britain, conducted on April 1, 2011. We multiplied this by 3.5 to estimate person-years at-risk during case ascertainment. Data were stratified by: age, sex, SES, and ethnicity (10-category), or by; age, sex, ethnicity (5-category), and age-at-immigration/generation status, depending on the availability of stratified Census data for each analysis.

### Exposure and Confounder Variables

Sociodemographic data including birthdate, sex, ethnicity, birth country, date of immigration to the United Kingdom, main or last occupation and postcode at initial referral were routinely collected at first contact. Participant ethnicity was self-ascribed to 1 of 18 categories in the 2011 census, from which we created 10- and 5-category variables for analyses (Appendix 1), guided by previous research.^7,11^ Generation status, based on birth country, was categorized as: white British (UK-born), white British (but born overseas), first-generation BME groups (ie, foreign-born), and second- or later-generation BME groups (ie, UK-born). For first-generation migrants, we categorized age-at-immigration as: migration during infancy (0–4 years old), childhood (5–12 years), adolescence (13–19 years), and adulthood (20+ years). We obtained confounder data on age (16–24, 25–29, 30–35), sex and a 6-category SES variable, based on occupation, classified according to official national guidance (Appendix 1).^25,26^ We geocoded participants to the small area neighborhood in which they lived at first referral, based on 530 “electoral wards” in the catchment area (median total population: 3992; interquartile range: 2426–5935). We estimated population density in people per square mile [ppsqm], based on 2011 Census data^22^ to create a 4-category equal-interval variable (48–4000; 4001–8000; 8001–12000; over 12000 ppsqm) to control for confounding. To inspect whether variation in risk by BME status was present in rural and urban areas, we used a binary version of this variable (less/more than 8000 ppsqm), which differentiated rural areas (small market towns, villages, and agricultural areas) from major towns and cities, as previously shown.^22^ We also controlled for confounding by multiple deprivation, with neighborhoods classified into 4 equal-interval groups, based on the proportion of households living in multiple deprivation in the 2011 Census (7.8–18.0%; 18.1–28.0%; 28.1–38.0%; 38.1–47.1%) (see Appendix 1 for more details).

### Statistical Analyses

We first reported descriptive characteristics of the sample. Next, we conducted 4 sets of multivariable Poisson analyses to test our hypotheses. First, we estimated unadjusted incidence rate ratios (IRR) for FEP by 10-category ethnicity, with the white British population as the reference category, followed by incremental adjustment for (1) age, sex and their interaction; (2) SES, and; (3) population density and multiple deprivation. For this final adjustment, we used multilevel Poisson models with random intercepts to account for hierarchical data (individuals within neighborhoods), excluding 28 participants of “No Fixed Abode,” who could not be geocoded to a residential address at first referral. Second, we tested whether the effect of ethnicity on psychosis risk differed by rural–urban status, by testing for effect modification between these variables via a likelihood ratio test (LRT). Due to sample size considerations, we used 5-category ethnicity in these analyses. Third, we examined whether FEP rates varied by exposure to the index immigration event among BME groups, by comparing incidence in first- and later-generation groups with the white British population. We tested whether patterns of psychosis risk by generation status also varied by 5-category ethnicity, via LRT as before. Finally, we investigated whether age-at-immigration was associated with FEP incidence, relative to the white British population, after adjustment for age and sex, and tested whether these patterns varied by ethnicity (5-category), as before. To investigate whether any observed age-at-immigration effects may have been due to time in the United Kingdom since migration, we followed the approach by Veling et al^19^ to test whether age-at-immigration was associated with psychosis risk in younger (16–24 years) and older (25–35 years) participants in a sensitivity analysis. If age-at-immigration was a stronger determinant of psychosis risk than time-in-the-UK, any signal for elevated psychosis risk in a specific age-at-immigration category should have been present in both our younger and older cohorts. Results for our main outcome (all FEP) are presented in the main text and tables. Given that 83.4% of the sample received a diagnosis of nonaffective psychosis, results for nonaffective psychosis, schizophrenia (50.9%) and affective psychoses (9.5%) are presented as supplemental material. Analyses were conducted using Stata (version 13).

### Ethics

Ethical approval was granted by Cambridgeshire III Local Research Ethics Committee (09/H0309/39).

## Results

### Baseline Characteristics

We identified 687 people with FEP during 2 million person-years at-risk, of which 50.9% met diagnostic criteria for schizophrenia (table 1). Detailed baseline characterization of the sample has been published,^22^ but briefly, two-thirds of FEP participants were men (*N* = 459; 66.8%), with younger age groups and lower SES groups over-represented (table 1). One quarter of FEP participants (*N* = 173; 25.2%) self-ascribed to a BME group, compared with 19.7% of the population at-risk (χ^2^ test: 13.0; *P* < .001). Of these, most FEP participants came from non-British white (9.9%), mixed (4.1%), black African (3.3%), or Pakistani (2.5%) backgrounds. Compared with the population at-risk, a greater proportion of FEP participants were of second- or later-generation status, had migrated before 20 years old and lived in urban areas (all *P* < .001, table 1). Median age-at-immigration in first-generation FEP participants (*N* = 103) was 20.1 years old (interquartile range [IQR]: 15.6–23.7) and was negatively correlated (corr = −0.72; *P* < .001; see supplementary figure 1) with years in the United Kingdom (median: 4.9 years; IQR: 2.6–9.5), although these patterns varied by ethnic minority group (*P* = .03; supplementary figure 1).

**Table 1.** Basic Sample Characteristics

Variable (Test; *P*-Value)^a^	Cases		Person-Years at-Risk		Crude Incidence	
	*N*	(%)	*N*	(%)	Rate	(95% CI)
Diagnosis (ICD-10)						
Any FEP (F10–33)	687	(100.0)	2021663	(100.0)	34.0	(31.5–36.6)
Schizophrenia (F20)	350	(50.9)	2021663	(100.0)	17.3	(15.6–19.2)
Other nonaffective psychosis (F21–29)	223	(32.5)	2021663	(100.0)	11.1	(9.7–12.6)
Bipolar disorder (F30–31)	65	(9.5)	2021663	(100.0)	3.2	(2.5–4.1)
Psychotic depression (F32–33)	19	(2.8)	2021663	(100.0)	0.9	(0.6–1.5)
Substance-induced psychoses (F10–19)	30	(4.4)	2021663	(100.0)	1.5	(1.0–2.1)
Ethnicity (χ^2^-test: 13.0; *P* < .001)^b^						
White, British	514	(74.8)	1623 285	(80.3)	31.7	(29.0–34.5)
White, non-British	68	(9.9)	207098	(10.2)	32.8	(25.9–41.6)
Indian	2	(0.3)	27911	(1.4)	7.2	(1.8–28.7)
Pakistani	17	(2.5)	20125	(1.0)	84.5	(52.5–135.9)
Bangladeshi	6	(0.9)	8401	(0.4)	71.4	(32.1–159.0)
Arabic ethnicities	4	(0.6)	4848	(0.2)	82.5	(31.0–219.8)
Black, African	23	(3.3)	17177	(0.8)	133.9	(89.0–201.5)
Black, Caribbean	10	(1.5)	5966	(0.3)	167.6	(90.2–311.5)
Mixed ethnicities	28	(4.1)	44013	(2.1)	63.6	(43.9–92.1)
Other ethnicities	15	(2.2)	62851	(3.1)	23.9	(14.4–39.6)
Generation status (FE-test; *P* < .001)						
White, British (UK-born)	511	(74.4)	1573700	(77.8)	32.5	(29.8–35.4)
White, British (born overseas)	3	(0.4)	49331	(2.4)	6.1	(2.0–18.9)
BME, later generation (UK-born)	67	(9.8)	73586	(3.6)	91.0	(71.7–115.7)
BME, first-generation (foreign-born)	106	(15.4)	325046	(16.1)	33.8	(27.0–39.4)
Age-at-immigration^c^ (FE-test: *P* < .001)						
0–4 years (infancy)	4	(3.9)	9505	(2.9)	42.1	(15.8–112.1)
5–12 years (childhood)	16	(15.5)	18322	(5.6)	87.3	(53.5–142.5)
13–19 years (adolescence)	31	(30.1)	70364	(21.6)	44.1	(31.0–62.6)
20+ years (adulthood)	52	(50.5)	226854	(69.8)	22.9	(17.5–30.1)
Rural–urban status^d^ (χ^2^-test: 26.4; *P* < .001)						
White British—rural	339	(51.4)	1216693	(60.2)	27.9	(25.0–31.0)
BME groups—rural	83	(12.6)	231999	(11.5)	35.8	(28.9–44.4)
White British—urban	156	(23.7)	406592	(20.1)	38.4	(32.8–44.9)
BME groups—urban	81	(12.3)	166510	(8.2)	48.6	(39.1–60.5)

*Note*: FEP, first-episode psychosis; FE-test, Fisher’s Exact test; BME, black and minority ethnic group.

^a^Test of whether the distribution of FEP participants is the same as the population at-risk, by each variable.

^b^χ^2^-test of white British vs all BME groups. A Fisher’s Exact test would not converge on 11 categories.

^c^“BME, first-generation” group only, excluding 3 participants missing age-at-immigration data (*N* = 103).

^d^Rural: 0–8000 people per square mile; Urban: 8001–22000 people per square mile.

### Incidence Rates by Ethnic Group

Crude FEP incidence rates were raised across most ethnic minority groups relative to the white British population (table 2); this pattern held for most diagnostic outcomes (supplementary figure 2). Progressive adjustment for age, sex, their interaction, SES, and neighborhood-level deprivation and population density did not substantially attenuate IRR estimates. Thus, after full adjustment (table 2), rates remained significantly elevated among people of black African (IRR: 4.06; 95% confidence interval [CI]: 2.63–6.25), black Caribbean (IRR: 4.63; 95% CI: 2.38–8.98), Pakistani (IRR: 2.31; 95% CI: 1.35–3.94), and mixed ethnic backgrounds (IRR: 1.71; 95% CI: 1.15–2.54). There was no evidence that risk by ethnic group differed for men and women (LRT for interaction χ^2^ on 9 *df*: 13.4, *P* = .15). These patterns were similar for nonaffective psychoses and schizophrenia separately (supplementary table 1). For schizophrenia, we observed substantially elevated rates across several ethnic groups after control for confounders, including Bangladeshi (IRR: 2.85; 95% CI: 1.06–7.67) and Arabic groups (IRR: 3.14; 95% CI: 1.00–9.85), albeit in a small sample of the latter group (N-4). People of Pakistani, black Caribbean, and mixed ethnic backgrounds were also at substantially elevated rates of affective psychoses (supplementary table 1) after adjustment. We found no evidence to suggest people of non-British white (IRR: 1.00; 95% CI: 0.77–1.32) or Indian (IRR: 0.29; 95% CI: 0.07–1.15) ethnicities were at elevated FEP risk overall (table 2).

**Table 2.** First-Episode Psychosis Incidence Rate Ratios by Ethnic Group, After Multivariable Adjustment

Ethnicity	Unadjusted		Adjustment 1		Adjustment 2		Adjustment 3^a^	
	IRR	(95% CI)	IRR	(95% CI)	IRR	(95% CI)	IRR	(95% CI)
Sample, *N*	687		687		687		659	
White, British	Ref		Ref		Ref		Ref	
White, non-British	1.00	(0.78–1.30)	1.21	(0.94–1.56)	1.11	(0.86–1.43)	1.00	(0.77–1.32)
Indian	**0.23**	**(0.06–0.94)**	0.27	(0.07–1.09)	0.28	(0.07–1.13)	0.29	(0.07–1.15)
Pakistani	**2.77**	**(1.71–4.49)**	**2.93**	**(1.80–4.74)**	**2.67**	**(1.65–4.34)**	**2.31**	**(1.35–3.94)**
Bangladeshi	**2.34**	**(1.05–5.24)**	**2.34**	**(1.05–5.23)**	2.19	(0.98–4.89)	2.03	(0.90–4.58)
Arabic ethnicities	**2.71**	**(1.01–7.25)**	2.51	(0.94–6.71)	2.26	(0.84–6.05)	2.16	(0.80–5.84)
Black, African	**4.20**	**(2.74–6.43)**	**4.62**	**(3.04–7.01)**	**4.21**	**(2.77–6.41)**	**4.06**	**(2.63–6.25)**
Black, Caribbean	**4.94**	**(2.56–9.55)**	**5.16**	**(2.76–9.66)**	**4.94**	**(2.64–9.24)**	**4.63**	**(2.38–8.98)**
Mixed ethnicities	**2.01**	**(1.37–2.94)**	**1.89**	**(1.29–2.77)**	**1.82**	**(1.24–2.66)**	**1.71**	**(1.15–2.54)**
Other ethnicities	0.78	(0.47–1.31)	0.77	(0.46–1.29)	0.73	(0.43–1.22)	0.75	(0.45–1.26)

*Note*: IRR, incidence rate ratio; 95% CI, 95% confidence interval. Bold denotes *P* < .05. Adjustment 1: Adjusted for age group, sex, and their interaction. Adjustment 2: Adjustment 1 + socioeconomic status + EIP setting. Adjustment 3: Adjustment 2 + population density + multiple deprivation.

^a^Twenty-eight participants of no fixed abode were excluded as they could not be assigned neighborhood-level exposures.

### Incidence Rates by Ethnic Group and Rural–Urban Status

There was no evidence that FEP risk by ethnicity differed between rural and urban populations (LRT *P* value for interaction: χ^2^ on 9 *df*: 13.4, *P* = .32). Thus, rates were raised for black, Pakistani, and Bangladeshi groups in both rural and urban regions, after adjustment for age, sex, SES, and neighborhood-level deprivation (table 3). There was no evidence that rates were raised for non-British white or other ethnic groups in rural or urban regions. These patterns were similar for nonaffective psychoses and schizophrenia (supplementary table 2). Although the sample of affective psychoses was small, there was evidence some ethnic minority groups (Pakistani and Bangladeshi, non-British white and “other” ethnicities) in rural areas had elevated risk (supplementary table 2).

**Table 3.** Rural–Urban Patterns in First-Episode Psychosis Incidence Rate Ratios by Ethnic Group

Broad ethnicity	Rural^a^				Urban^a^			
	*N*	%	aIRR	(95% CI)	*N*	%	aIRR	(95% CI)
White, British	339	80.3	Ref		156	65.8	Ref	
White, non-British	33	7.8	1.03	(0.71–1.48)	30	12.7	1.01	(0.68–1.50)
Black ethnicities	12	2.8	**3.19**	**(1.79–5.70)**	19	8.0	**5.18**	**(3.20–8.37)**
Pakistani and Bangladeshi	28	6.6	**3.08**	**(1.62–5.85)**	19	8.0	**2.01**	**(1.10–3.66)**
Other ethnicities	10	2.4	1.29	(0.88–1.91)	13	5.5	0.84	(0.52–1.37)
LRT *P* value for interaction	.32							

*Note*: aIRR, adjusted incidence rate ratio for age group, sex, their interaction, SES, and neighborhood-level multiple deprivation. Bold denotes *P* ≤ .05.

^a^Based on a dichotomous cut-off of 8000 people per square mile, corresponding to the distinction between rural areas and major towns and cities in the catchment area.

### Incidence Rates by Generation Status

**Fig. 1.**
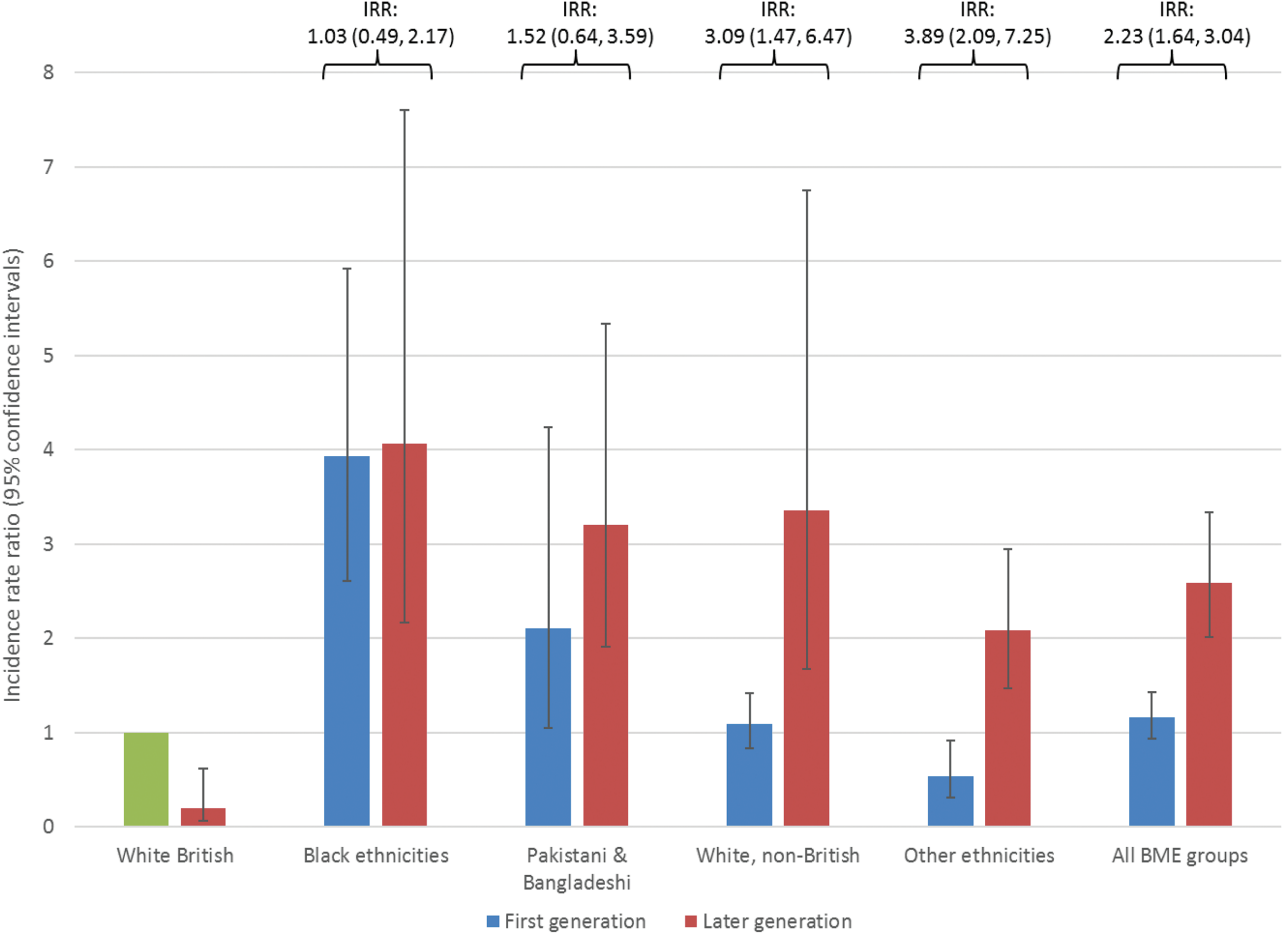
Incidence rate ratios for first-episode psychosis (FEP) by generation status and broad ethnic group. Overall there was evidence that FEP risk by generation status varied by ethnic group (LRT-χ^2^*P*-value for interaction between ethnicity and generation status on 3 degrees of freedom: χ^2^ = 9.2; *P* = .03). Thus, compared with the UK-born white British group, rates were raised to a similar extent for first- and later-generation black and Pakistani and Bangladeshi groups, with no statistically significant differences in risk by generation (supplementary table 3). For non-British white and other ethnic groups, excess rates were confined to later-generation groups. Foreign-born white British groups and first generation “other” ethnic groups were at significantly reduced psychosis risk compared with the UK-born white British group. All incidence rate ratios are adjusted for age and sex. The white British (UK-born) reference population is shown in green, with the white British (born overseas) shown in red. BME: black and minority ethnic. Data corresponding to this figure are presented in supplementary table 3.

Second- and later-generation (ie, UK-born) BME groups were at increased FEP risk relative to the UK-born white British population, after adjustment for age and sex (figure 1 and supplementary table 3; IRR: 2.59; 95% CI: 2.01–3.34). While there was no overall evidence of increased rates in first-generation migrants (IRR: 1.16, 95% CI: 0.94–1.43), this pattern varied by ethnicity (LRT-χ^2^ on 3 *df*: 9.2, *P* = .03; figure 1, supplementary table 3). Thus, rates were elevated to a similar extent for first- and later-generation black and Pakistani and Bangladeshi groups (figure 1, supplementary table 3), after adjustment for age and sex, compared with the UK-born white British population. By contrast, while first generation non-British white migrants were not elevated (IRR: 1.09; 95% CI: 0.83–1.42), we observed excess rates in the small proportion of later-generation (ie, UK-born) non-British white migrants (IRR: 3.36; 95% CI: 1.67–6.75).

### Incidence Rates by Age-at-Immigration

**Fig. 2.**
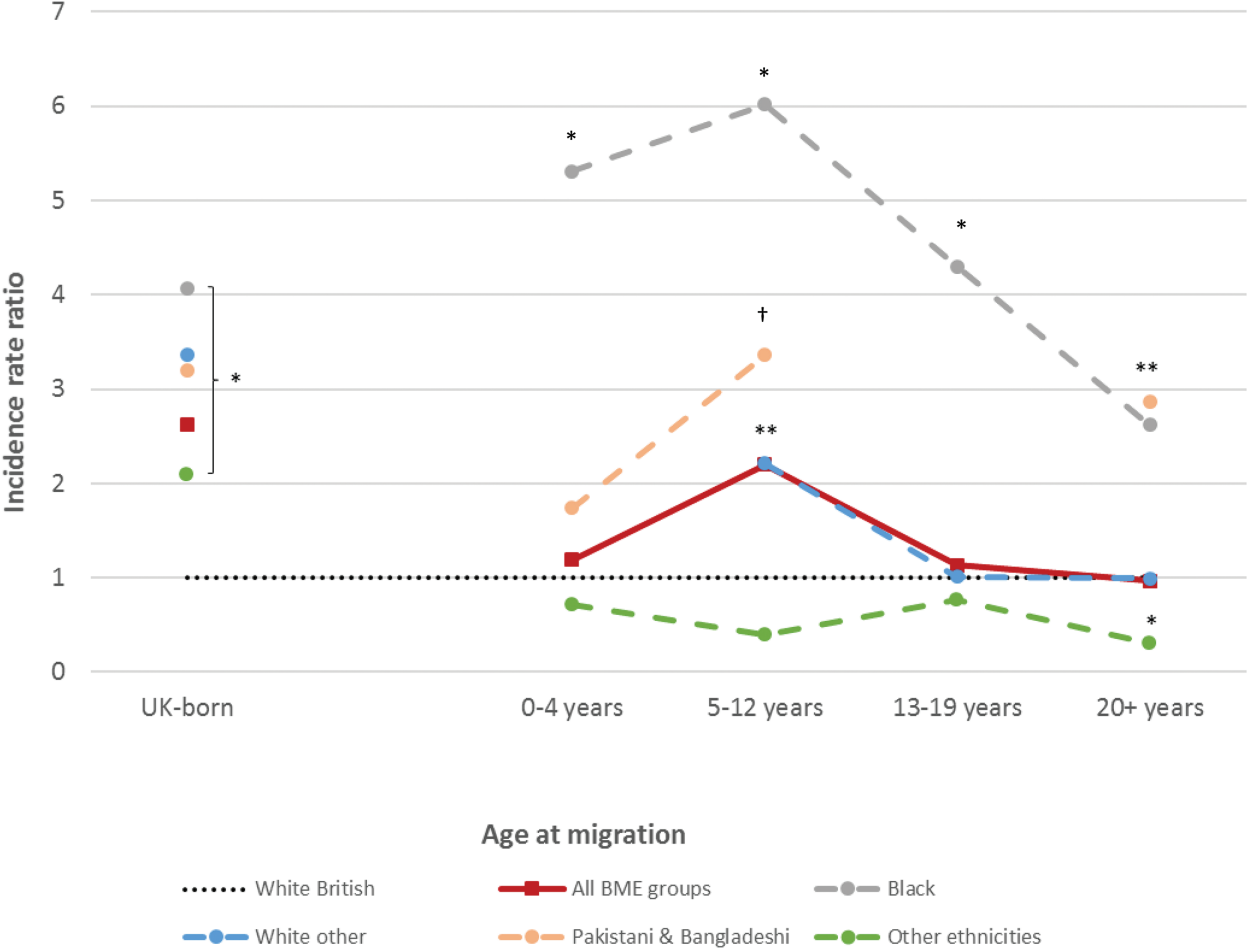
Incidence of all clinically relevant psychotic disorders by age-at-immigration, major ethnic group, and generation status. Incidence rate ratios (IRR) by age-at-immigration show a peak with childhood migration (5–12 years old) for all black and minority ethnic (BME) groups overall (IRR: 2.20; 95% CI: 1.33–3.62). There was no evidence that this effect differed by 5-category ethnicity (LRT χ^2^ on 16 *df*: 23.1; *P* = .11). This finding was independently replicated in first-generation black (IRR: 6.02; 95% CI: 2.69–13.47) and non-British white (IRR: 2.21; 95% CI: 1.05–4.68) immigrants, with a trend in this direction for Pakistani and Bangladeshi (IRR: 3.36; 95% CI: 0.84–13.49; ^†^*P* = .09). IRRs appeared to decrease in relation to later age-at-immigration. Only first-generation black (IRR: 2.62; 95% CI: 1.24–5.55) and Pakistani and Bangladeshi migrants (IRR: 2.87; 95% CI: 1.18–6.94) who moved to the United Kingdom in adulthood were at significantly increased psychosis risk compared with the white British population. People from the “other ethnicities” group who migrated aged 20 years or older were at significantly decreased risk of psychosis compared with the UK-born white British group (IRR: 0.31; 95% CI: 0.11–0.82). IRR for second- and later-generation UK-born groups are shown for comparison. There were insufficient first-episode psychosis participants of foreign-born white British descent (*n* = 3) to analyze results by age-at-immigration. All IRRs adjusted for age group and sex. 95% confidence intervals omitted for presentational purposes. *P < .05. ^†^*P* = .09.

Three (2.8%) first-generation FEP participants with missing data on immigration date were excluded from these analyses. For remaining participants (*N* = 103), those who immigrated to the United Kingdom in childhood (5–12 years old) experienced elevated rates of psychotic disorders compared with the UK-born white British population (IRR: 2.20; 95% CI: 1.33–3.62; figure 2), but there was no evidence that immigration at other ages was associated with FEP risk (supplementary table 4). Excess risk associated with childhood immigration was observed independently for first generation black (IRR: 6.02; 95% CI: 2.69–13.47) and non-British white groups (IRR: 2.21; 95% CI: 1.05–4.68), with a trend in this direction for Pakistani and Bangladeshi migrants (IRR: 3.36; 95% CI: 0.84–13.49; *P* = .09; figure 2). Black (IRR: 2.62; 95% CI: 1.24–5.55) and Pakistani and Bangladeshi (IRR: 2.87; 95% CI: 1.18–6.94) migrants who immigrated to the United Kingdom in adulthood (20+ years) also remained at increased psychosis risk (figure 2). Lower rates in adult migrants from “other” ethnic backgrounds (IRR: 0.31; 95% CI: 0.11–0.82) were also observed, but difficult to interpret given within-group heterogeneity. There was no evidence that these patterns differed between our younger and older cohorts (LRT-χ^2^ on 4 *df*: 6.0; *P* = 0.20; supplementary table 4), suggesting age-at-immigration operated independently of time-in-the-UK.

## Discussion

Our findings demonstrate that the incidence of all major psychotic disorders were raised in several ethnic minority groups after controlling for important confounders, including SES, neighborhood-level population density, and multiple deprivation (Hypothesis 1). Arising within a large, diverse, and representative population at-risk, elevated risks were largest and most consistent for people of black Caribbean, black African, and Pakistani origin. We extend previous knowledge by demonstrating that patterns of risk among BME groups were broadly similar for people living in rural or urban environments (Hypothesis 2). Rates were equivocally raised for first- and later-generation black Caribbean and African, Pakistani, and Bangladeshi groups (Hypothesis 3), though for non-British white minorities, elevated rates were restricted to UK-born, later generations. Finally, for first-generation migrants—exposed to the index migration event—excess risk was consistently most-pronounced in those migrating during childhood, partially supporting Hypothesis 4.

### Methodological Considerations

Incidence rates were based on incepted cases presenting to NHS mental health services in our catchment area, which may have differed slightly to the true incidence in the population. Nevertheless, we do not believe our methodology led to substantial under-ascertainment of FEP cases in our catchment, either nondifferentially, or differentially by ethnicity. EIP services were the sole referral point for any suspected psychosis presenting to multiple sources, actively engaged in outreach to minimize under ascertainment, and operated across rural and urban settings within our catchment area. Further, both our overall estimates of incepted incidence,^22^ and those by ethnicity here, are in line with previous rates from more urban populations,^1,3,27^ suggesting differential under-ascertainment in BME groups, which would have made our results conservative, was unlikely. We also believe that over-ascertainment of ethnic minority cases was an unlikely explanation of the excess incidence rates observed in our study. We cannot exclude the possibility that patterns of psychiatric help-seeking by generation status may have differed,^28^ but little empirical work on how this ultimately may affect case ascertainment has been conducted to date. We used a 2-stage diagnostic procedure established well-validated, research-based FEP diagnoses; clinical diagnoses obtained in stage one were provided by an ethnically diverse group of clinicians, ratified in stage 2 by independent (ie, different) diagnosticians using OPCRIT.

We controlled for several important confounders in our main analyses, including age, sex, SES, and neighborhood-level deprivation and population density. We could not control for confounders omitted from the Census, including paternal age, parental SES, substance use, or family history of mental illness. Control for participant rather than parental SES will have led to conservative IRR estimates in ethnic minority groups, given the possibility of downward social drift for some FEP participants. This was the largest epidemiological study of FEP conducted in the United Kingdom since the multi-center ÆSOP study,^27^ and our diverse study setting had a higher proportion of BME groups (19.7% vs 17.6%), although the ethnic composition of these samples differed substantially, with 52.0% of the BME population in our study of non-British white ethnicity (vs 9.9% in ÆSOP^27^). This resulted in fewer BME cases in our study overall, given the incidence in non-British white minorities, leading to broader confidence intervals when estimating FEP risk for some specific ethnic groups. We cannot exclude type II error as an explanation for some null findings, although the absence of substantially elevated risk in our largest BME group—non-British white migrants—should be robust. Furthermore, while FEP incidence appeared to peak for first-generation participants who immigrated in childhood, we could not determine whether this differed significantly from risk at other ages of immigration. Nonetheless, there was some replicability of this finding, observed in black, Pakistani and Bangladeshi and non-British white migrants independently.

The 2011 UK Census provided valid ethnicity categories^29^ and included measures to minimize, estimate, and adjust for nonresponse,^30^ making substantial under-estimation of our denominator population unlikely. The census included information on age-at-immigration for the first time, for which we obtained especially commissioned denominator data, additionally stratified by age, sex, and ethnicity. For this reason, our age-at-immigration categories were chosen a priori, as they had to pass stringent ONS disclosure benchmarks; therefore this data could not be additionally stratified by SES.

### Meaning of the Findings

Consistent with the previous literature,^1,3,7^ people of black African, black Caribbean, and Pakistani origin were 3–5 times more likely to be diagnosed with a psychotic disorder than the white British population. These risks were raised to a similar extent in rural and urban parts of our study region. Previous studies in rural settings have generally lacked sufficient ethnic diversity to investigate this issue.^31,32^ Our study overcomes this limitation. This is important because the selection processes which influence whether people live in more rural or urban areas may mean that the distribution of social, genetic, and environmental determinants of psychosis risk are stratified in the population. If this were the case, as suggested by proponents of the social drift hypothesis,^33,34^ it would imply that—on average—variation in psychosis risk would be less apparent in more rural populations; in contrast, we observed substantially elevated risk of several psychotic outcomes in BME groups living in rural areas, independent of SES and area-level deprivation. This implies that other social exposures, including putative roles for ethnic density,^35^ social isolation,^35^ discrimination,^36^ and stress experienced by BME groups operate across the entire rural–urban gradient of environmental susceptibility.

We found no evidence, overall, that non-British white groups were at elevated risk of FEP, including first-generation migrants. This is an important null finding, given the large number of white migrants, particularly from Eastern Europe, who have arrived in the United Kingdom since EU expansion in 2004. Nonetheless, we did observe some variation in risk within this group, including raised rates among non-British white minorities born in the United Kingdom. Although our sample was small, this group included a high proportion of people (3 of 8; 37.5%) who self-ascribed as “white Gypsy or traveler” in the 2011 census. While several explanations for this excess may exist, including under-enumeration of these communities in censuses, such groups may also be subject to high degrees of marginalization and discrimination; interestingly, our data replicate a similar finding in inner London among UK-born, non-British white minorities.^37^ We also reported a trend toward higher schizophrenia rates among people of Arabic origin, never previously studied in the United Kingdom. While this requires replication in a larger sample, the 2001–2011 intercensal period witnessed increased discrimination toward this diverse ethnic group,^38^ coinciding with the emergence of elevated schizophrenia risk in this group; from limited epidemiological evidence in Arabic countries, rates do not appear to be raised prior to migration.^39^

With regard to age-at-immigration and psychosis, 2 studies^9,40^ (one, an un-reviewed letter^40^), found no association in Denmark, but most migrants were from neighboring Scandinavian countries. In 2 more ethnically diverse studies, younger ages-at-migration predicted later psychosis risk among migrants,^6,19^ peaking with infant migration in one study^19^ and later childhood migration in another,^6^ partially consistent with our findings. We also hypothesized that migration during adolescence would increase psychosis risk, but found only limited evidence (ie, among older participants in our cohort, supplementary table 4). One possibility here is that younger participants in our cohort who migrated in adolescence might not, on average, have passed through a sufficient latency period to develop psychotic symptoms, suggesting that both age-at-immigration and time lived in the “host” country may jointly influence psychosis risk.

If early life migration does increase psychosis risk, it is worth considering the possible underlying mechanisms through which this may emerge. Sociocognitive models, eg, emphasize early childhood as an important window for developing Theory of Mind (the ability to attribute mental states to the self and others), acquiring complex language skills, and establishing social ties with nonkin peers.^41,42^ Migration during this period may disrupt typical neurodevelopment, particularly when the migration event is stressful or invokes substantial changes to language, peer group maintenance (eg, by changing schools^43^) or sociocultural adaptation. Sociocognitive impairments,^44,45^ social withdrawal,^46–48^ and social stress^49^ in childhood have been associated with later psychosis onset, while migration during childhood and early adolescence has also been associated with social withdrawal and perceived social support in adulthood.^50^ Corollary data from social neuroscience suggests that among healthy volunteers, second-generation BME participants exhibit elevated neural responses to social stress compared with native-born participants,^51^ although this requires replication in first-generation immigrants. Further research on the potential psychosocial stressors associated with migration during sensitive neurodevelopmental windows should elucidate clues to the elevated psychosis risk in some migrant groups. If true, interventions which promote social support and resilience during important periods of sociocultural and neurodevelopmental transitions in childhood may ameliorate such serious mental health outcomes later in life.^52^

## Supplementary Material

Supplementary data are found at *Schizophrenia Bulletin* online.

## Funding

This work was supported by a Sir Henry Wellcome Research Fellowship from the Wellcome Trust (WT085540 to J.B.K.), a Sir Henry Dale Fellowship jointly funded by the Wellcome Trust and the Royal Society (101272/Z/13/Z to J.B.K.) and by the National Institute of Health Research (RP-PG-0606-1335 to J.P.). Prof Peter Jones directs the NIHR Collaboration for Leadership in Applied Health Research and Care (CLAHRC) East of England.

## Supplementary Material

Supplementary_Material__d12_

## Appendix 1

### Detailed Methodology on Exposure Variables

We recorded detailed sociodemographic data on all people accepted for inclusion in EIP services consistent with the classification systems available for our denominator population, estimated from the 2011 Census. For ethnicity, we initially asked people to self-ascribe to one of 18 Census categories, from which we created 10- and 5-category ethnicity variables for analytical purposes. The 10-category ethnicity variable included: white British, non-British white ethnicities (white Irish, white traveller or gypsy, other white ethnicities), black African, black Caribbean, mixed ethnicities (mixed white and black Caribbean, mixed white and black African, mixed white and Asian, other mixed ethnicity), Indian, Pakistani, Bangladeshi, Arabic ethnicity and all other ethnicities (Chinese, other Asian, other black ethnicities, other). The five-category ethnicity included: white British, non-British white, black ethnicities (black African, black Caribbean, other black ethnicities), Pakistani and Bangladeshi, and all other ethnic groups (Indian, Chinese, other Asian, Arabic, mixed ethnicities, other ethnicities). Detailed data on country of birth and, if relevant, month and year of immigration to the UK were also ascertained from all participants at first contact with an EIP professional. Data on self-ascribed ethnicity and country of birth was obtained from all clients. Age-at-migration could not be ascertained for 3 participants.

We categorized participant SES according to National Statistics Socio-Economic Classification,^25,26^ based on occupation at first referral, 7 as: professional and managerial, intermediate occupations, self-employed, lower supervisory and technical occupations, semi-routine and routine occupations, and those in long-run unemployment, never worked or students.

We defined multiple deprivation according to 4 domains of deprivation used in the 2011 Census : unemployment (any working-age adult classified as long-term sick or unemployed), education (households without any adult with age 16 national qualifications (5 or more GCSEs) or without a full time student), health (any household member with bad or very bad self-rated health or a long-term limiting health problem) and the living environment (household overcrowding, no central heating, or more than one family sharing a single dwelling). For each neighbourhood, we estimated the proportion of households who were classified as deprived on at least 2 of these 4 domains, and categorized this into 4 equal interval bands, as described in the main article.
