# Supplementary material for: Ethnic Minority Status, Age-at-Immigration and Psychosis Risk in Rural Environments: Evidence From the SEPEA Study
Source: Schizophr Bull. 2017 May 17;43(6):1251–61. doi: 10.1093/schbul/sbx010 (PMC5737276; doi:10.1093/schbul/sbx010)
Supplement: Supplementary_Material__d12_ [file sbx010_suppl_supplementary_material__d12_.doc]

**Appendix 1: Detailed methodology on exposure variables**

We recorded detailed sociodemographic data on all people accepted for inclusion in EIP services consistent with the classification systems available for our denominator population, estimated from the 2011 Census. For ethnicity, we initially asked people to self-ascribe to one of 18 Census categories, from which we created 10- and 5-category ethnicity variables for analytical purposes. The ten-category ethnicity variable included: white British, non-British white ethnicities (white Irish, white traveller or gypsy, other white ethnicities), black African, black Caribbean, mixed ethnicities (mixed white and black Caribbean, mixed white & black African, mixed white & Asian, other mixed ethnicity), Indian, Pakistani, Bangladeshi, Arabic ethnicity and all other ethnicities (Chinese, other Asian, other black ethnicities, other). The five-category ethnicity included: white British, non-British white, black ethnicities (black African, black Caribbean, other black ethnicities), Pakistani and Bangladeshi, and all other ethnic groups (Indian, Chinese, other Asian, Arabic, mixed ethnicities, other ethnicities). Detailed data on country of birth and, if relevant, month and year of immigration to the UK were also ascertained from all participants at first contact with an EIP professional. Data on self-ascribed ethnicity and country of birth was obtained from all clients. Age-at-migration could not be ascertained for three participants.

We categorized participant SES according to National Statistics Socio-Economic Classification,^25,26^ based on occupation at first referral,7 as: professional & managerial, intermediate occupations, self-employed, lower supervisory & technical occupations, semi-routine & routine occupations, and those in long-run unemployment, never worked or students.

We defined multiple deprivation according to 4 domains of deprivation used in the 2011 Census : unemployment (any working-age adult classified as long-term sick or unemployed), education (households without any adult with age 16 national qualifications (5 or more GCSEs) or without a full time student), health (any household member with bad or very bad self-rated health or a long-term limiting health problem) and the living environment (household overcrowding, no central heating or more than one family sharing a single dwelling). For each neighbourhood we estimated the proportion of households who were classified as deprived on at least 2 of these 4 domains, and categorised this into 4 equal interval bands, as described in the main paper.

**Supplementary Figure 1 [SF1]: Correlation and median age-at-migration and time in the UK before first referral in first-generation FEP participants, by 5-category ethnicity**

**
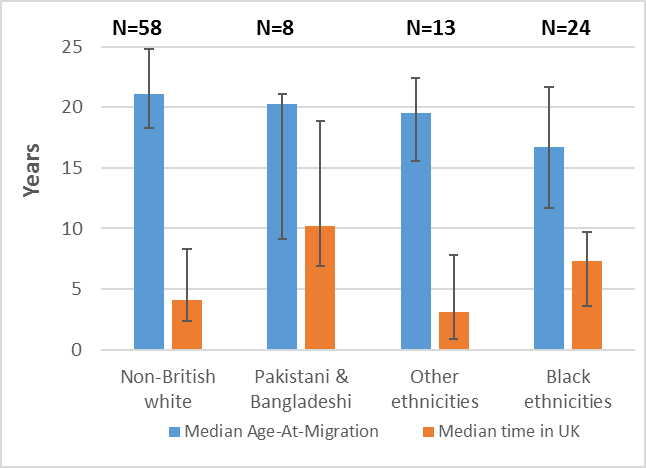

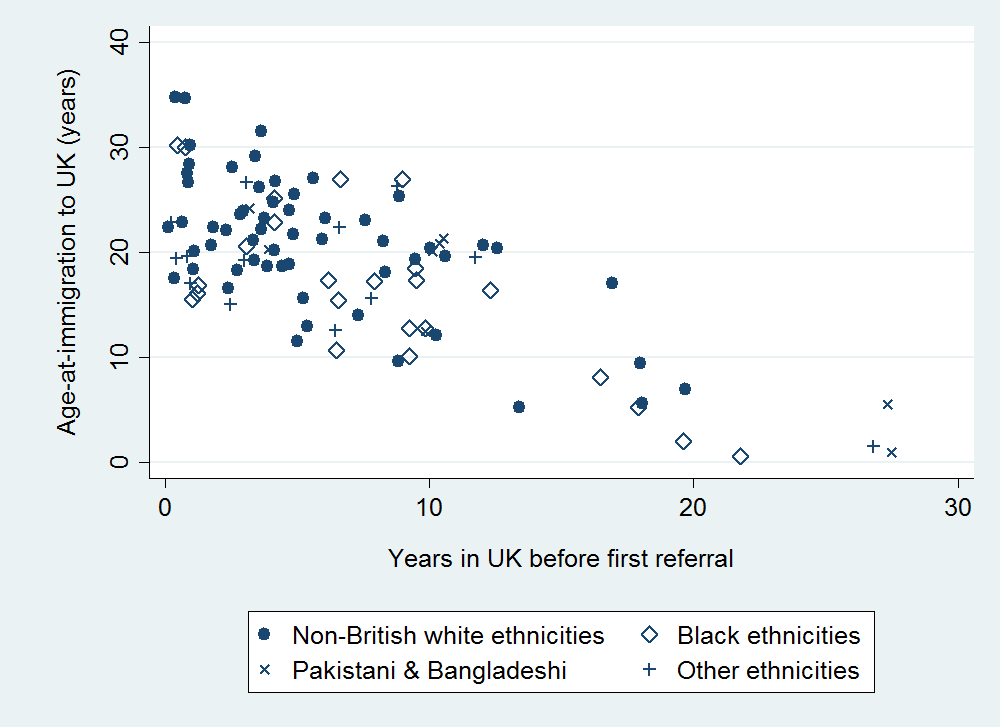
**

**A**

**B**

**Figure legend: A.** Correlation (-0.72; p<0.001) between age-at-migration and years in the UK before first referral in first generation FEP participants, coded by major (5-category) ethnic group (foreign-born white British FEP participants, n=3, not shown). **B.** Median age-at-migration and time lived in the UK before FEP referral, by major (5-category) ethnic group (foreign-born white FEP participants not shown)**.** Error bars represented interquartile ranges [IQR]. There was weak evidence that median age-at-migration (median test *χ*^2^ on 3*df*: 7.5; p=0.06) and median time in the UK before FEP referral (median test *χ*^2^ on 3*df*: 7.2; p=0.07) differed between ethnic groups.

**Supplementary Figure 2 [SF2]: Crude incidence of all clinically relevant psychotic disorders by major ethnic group and diagnostic category**


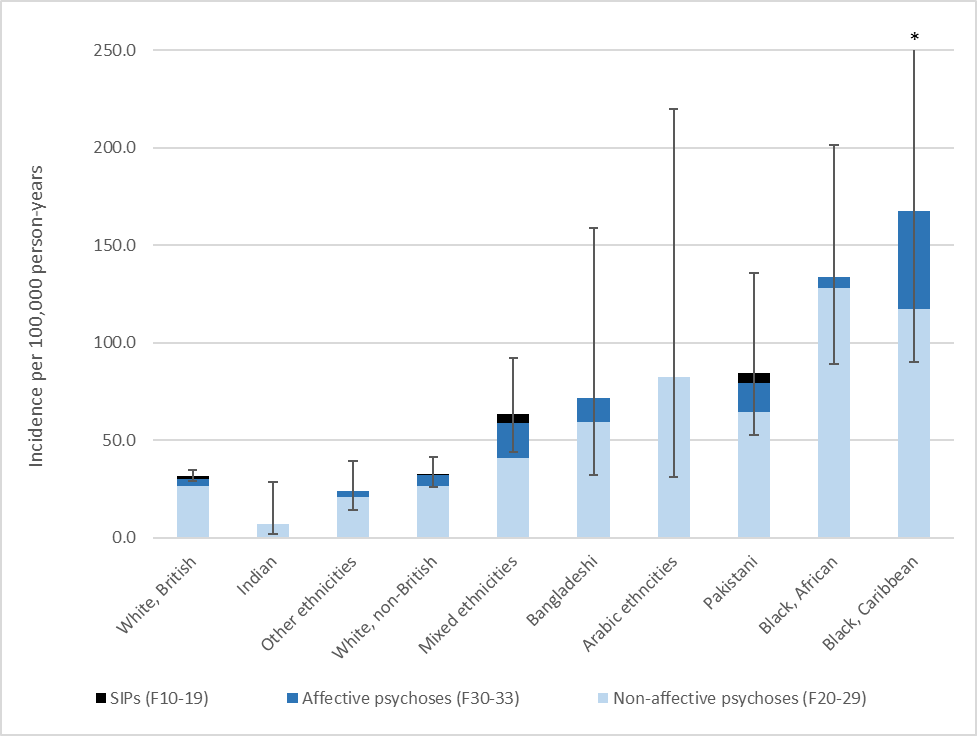


**Legend:** Crude incidence rates and 95% confidence intervals for all clinically-relevant psychotic disorders (F10-33) were raised in several ethnic groups relative to the white British group, except people of Indian, non-British white or “other” ethnicities. Rates appear to be highest for people of black African and Caribbean origin. Inspection of data by broad diagnostic category suggests these patterns are apparent for non-affective and affective psychoses independently.

*The upper 95% confidence limit for the black Caribbean group was truncated for presentational purposes (upper bound = 311.5)

Supplementary Table 1 [ST1]: Adjusted incidence rate ratios of specific psychotic outcomes by major ethnic group

|  | **Non-affective psychoses [F20-29]** | | **Schizophrenia [F20]** | | **Affective psychoses**  **[F30-33]** | |
| --- | --- | --- | --- | --- | --- | --- |
|  | aIRR^1^ | (95% CI) | aIRR^1^ | (95% CI) | aIRR^1^ | (95% CI) |
| White, British | Ref |  | Ref |  | Ref |  |
| White, non-British | 1.05 | (0.79, 1.40) | 1.06 | (0.74, 1.54) | 1.86 | (0.98, 3.51) |
| Indian | 0.33 | (0.08, 1.33) | 0.55 | (0.14, 2.23) | - |  |
| Pakistani | **2.42** | **(1.39, 4.20)** | **3.41** | **(1.86, 6.25)** | **4.68** | **(1.45, 15.04)** |
| Bangladeshi | 2.15 | (0.89, 5.20) | **2.85** | **(1.06, 7.67)** | 3.71 | (0.51, 26.90) |
| Arabic ethnicities | 2.65 | (0.99, 7.11) | **3.14** | **(1.00, 9.85)** | - |  |
| Black, African | **4.73** | **(3.07, 7.28)** | **4.62** | **(2.64, 8.10)** | 1.87 | (0.26, 13.54) |
| Black, Caribbean | **4.07** | **(1.93, 8.59)** | **3.74** | **(1.39, 10.05)** | **15.05** | **(4.69, 48.28)** |
| Mixed ethnicities | 1.38 | (0.86, 2.22) | 1.97 | (0.81, 4.79) | **5.08** | **(2.41, 10.70)** |
| Other ethnicities | 0.74 | (0.43, 1.29) | 0.66 | (0.31, 1.40) | 0.99 | (0.24, 4.10) |

**Bold** denotes p≤0.05

aIRR: Adjusted incidence rate ratio; 95% CI: 95% confidence interval

^1^Incidence rate ratios adjusted for age group, sex, their interaction and socioeconomic status

**Supplementary Table 2 [ST2]: Incidence rates of selected psychotic disorders** by ethnic group and rural-urban status

|  | **Rural†** | | | | **Urban†** | | | |
| --- | --- | --- | --- | --- | --- | --- | --- | --- |
| **Broad ethnicity** | **N** | **%** | **aIRR** | **(95% CI)** | **N** | **%** | **aIRR** | **(95% CI)** |
| *Non-affective psychosis (F20-29)* |  |  |  |  |  |  |  |  |
| White, British | 293 | 80.3 | Ref |  | 125 | 65.8 | Ref |  |
| White, non-British | 25 | 7.8 | 0.89 | (0.59, 1.34) | 25 | 12.7 | 1.04 | (0.67, 1.61) |
| Black ethnicities | 12 | 2.8 | **3.63** | **(2.03, 6.50)** | 15 | 8.0 | **5.00** | **(2.92, 8.58)** |
| Pakistani & Bangladeshi | 8 | 6.6 | **2.91** | **(1.43, 5.92)** | 10 | 8.0 | 1.92 | (0.97, 3.78) |
| Other ethnicities | 20 | 2.4 | 1.06 | (0.67, 1.67) | 15 | 5.5 | 0.82 | (0.48, 1.42) |
| **LRT p-value for interaction:** | 0.69 |  |  |  |  |  |  |  |
|  |  |  |  |  |  |  |  |  |
| *Schizophrenia (F20)* |  |  |  |  |  |  |  |  |
| White, British | 168 | 81.2 | Ref |  | 77 | 64.2 | Ref |  |
| White, non-British | 14 | 6.8 | 0.87 | (0.50, 1.51) | 15 | 12.5 | 1.01 | (0.58, 1.78) |
| Black ethnicities | 6 | 2.9 | **3.09** | **(1.36, 7.02)** | 10 | 8.3 | **5.32** | **(2.74, 10.32)** |
| Pakistani & Bangladeshi | 6 | 2.9 | **3.67** | **(1.60, 8.39)** | 9 | 7.5 | **2.67** | **(1.28, 5.58)** |
| Other ethnicities | 13 | 6.3 | 1.19 | (0.67, 2.11) | 9 | 7.5 | 0.78 | (0.39, 1.57) |
| **LRT p-value for interaction:** | 0.62 |  |  |  |  |  |  |  |
|  |  |  |  |  |  |  |  |  |
| *Affective psychosis (F30-33)*^‡^ |  |  |  |  |  |  |  |  |
| White, British | 32 | 66.7 | Ref |  | 21 | 60.0 | Ref |  |
| White, non-British | 7 | 14.6 | **2.32** | **(1.00, 5.40)** | 5 | 14.3 | 1.33 | (0.49, 3.63) |
| Black ethnicities | 0 | 0.0 | - | - | 4 | 11.4 | **9.56** | **(3.23, 28.32)** |
| Pakistani & Bangladeshi | 2 | 4.2 | **3.47** | **(1.50, 8.02)** | 2 | 5.7 | 1.11 | (0.32, 3.77) |
| Other ethnicities | 7 | 14.6 | **5.12** | **(1.10, 23.85)** | 3 | 8.6 | 2.54 | (0.57, 11.32) |
| **LRT p-value for interaction:** | N/A |  |  |  |  |  |  |  |
|  |  |  |  |  |  |  |  |  |
|  |  |  |  |  |  |  |  |  |

**Bold** denotes p≤0.05

aIRR: adjusted incidence rate ratio for age group, sex, SES and neighborhood-level multiple deprivation

^†^Based on a dichotomous cut-off of 8,000 people per square mile, corresponding to the distinction between rural areas and major towns and cities in the catchment area

^‡^Results presented from stratified analyses by rural-urban status. Formal test of statistical interaction between ethnicity and rural-urban status was not reported because a Poisson regression model fitted with the interaction term would not converge

**Supplementary Table 3 [ST3]**: Adjusted incidence rate ratios of various psychotic disorders by generation status and broad ethnic group

| **Generation status by broad ethnic group** | **Denominator** | **All FEP [F10-33]** | | **Non-affective psychoses [F20-29]** | | **Affective psychoses**  **[F30-33]** | |
| --- | --- | --- | --- | --- | --- | --- | --- |
|  | Person-years (%) | aIRR^1^ | (95% CI) | aIRR^1^ | (95% CI) | aIRR^1^ | (95% CI) |
| **White, British (UK-born)** | 1,573,700 (77.8) | Ref |  | Ref |  | Ref |  |
| **All ethnicities:** |  |  |  |  |  |  |  |
| White, British (Born overseas) | 49,331 (2.4) | **0.20** | **(0.06, 0.62)** | **0.16** | **(0.04, 0.63)** | 0.63 | (0.09, 4.57) |
| First-generation BME | 325,046 (16.1) | 1.16 | (0.94, 1.43) | 1.17 | (0.93, 1.47) | 1.52 | (0.85, 2.72) |
| Later generation BME | 73,586 (3.6) | **2.59** | **(2.01, 3.34)** | **2.24** | **(1.67, 3.02)** | **5.64** | **(3.18, 10.02)** |
|  |  |  |  |  |  |  |  |
| **Broad ethnic group:** |  |  |  |  |  |  |  |
| *Black ethnicities* | *28,020 (1.4)* |  |  |  |  |  |  |
| First-generation | 21,227 (1.0) | **3.93** | **(2.61, 5.92)** | **4.24** | **(2.76, 6.51)** | 3.14 | (0.76, 12.91) |
| Later generation | 6,793 (0.3) | **4.06** | **(2.17, 7.60)** | **3.83** | **(1.90, 7.71)** | **8.19** | **(1.99, 33.62)** |
| Later vs. first-generation |  | 1.03 | (0.49, 2.17) | 0.90 | (0.40, 2.03) | 2.61 | (0.37, 18.54) |
| *Pakistani & Bangladeshi* | *28,327 (1.4)* |  |  |  |  |  |  |
| First-generation | 14,704 (0.7) | **2.11** | **(1.05, 4.24)** | 1.86 | (0.83, 4.17) | **4.99** | **(1.21, 20.57)** |
| Later generation | 13,623 (0.7) | **3.20** | **(1.91, 5.34)** | **3.04** | **(1.71, 5.39)** | 4.07 | (0.99, 16.71) |
| Later vs. first-generation |  | 1.52 | (0.64, 3.59) | 1.63 | (0.61, 4.36) | 0.82 | (0.11, 5.83) |
| *White, non-British* | *207,953 (10.2)* |  |  |  |  |  |  |
| First-generation | 201,006 (9.9) | 1.09 | (0.83, 1.42) | 1.05 | (0.78, 1.42) | 1.65 | (0.83, 3.26) |
| Later generation | 6,947 (0.3) | **3.36** | **(1.67, 6.75)** | **2.97** | **(1.33, 6.66)** | **8.23** | **(2.00, 33.77)** |
| Later vs. first-generation |  | **3.09** | **(1.47, 6.47)** | **2.83** | **(1.21, 6.62)** | **4.99** | **(1.09, 22.93)** |
| *Other ethnicities* | *134,331 (6.6)* |  |  |  |  |  |  |
| First-generation | 88,110 (4.4) | **0.54** | **(0.31, 0.91)** | 0.59 | (0.34, 1.02) | 0.37 | (0.05, 2.65) |
| Later generation | 46,221 (2.3) | **2.08** | **(1.47, 2.95)** | **1.67** | **(1.10, 2.54)** | **5.35** | **(2.64, 10.85)** |
| Later vs. first-generation |  | **3.89** | **(2.09, 7.25)** | **2.85** | **(1.44, 5.62)** | **14.58** | **(1.84, 115.29)** |
| LRT-χ^2^ p-value for interaction between ethnicity & generation status (3 degrees of freedom) |  | χ^2^=9.2 | p=0.03 | χ^2^=5.8 | p=0.12 | χ^2^=4.6 | p=0.20 |

**Bold** denotes p≤0.05

BME: black and minority ethnic; aIRR: adjusted incidence rate ratios; 95% CI: 95% confidence intervals

^1^Adjusted for age group, sex and their interaction

**Supplementary Table 4 [ST4]: Relationship between FEP rates and age-at-migration, stratified by age-at-referral^1^**

| **Age-at-migration** | **All ages (16-35 years)** | | | |  | **16-24 years old** | | | |  | **25-35 years old** | | | |
| --- | --- | --- | --- | --- | --- | --- | --- | --- | --- | --- | --- | --- | --- | --- |
|  | **N** | **%** | **aIRR** | **(95% CI)** |  | **N** | **%** | **aIRR** | **(95% CI)** |  | **N** | **%** | **aIRR** | **(95% CI)** |
| White, British (UK-born) | 511 | (87.9) | Ref |  |  | 348 | (87.9) | Ref |  |  | 163 | (74.8) | Ref |  |
| 0-4 years | 4 | (0.5) | 1.19 | (0.44, 3.17) |  | 2 | (0.5) | 0.78 | (0.19, 3.13) |  | 2 | (0.9) | 2.42 | (0.60, 9.78) |
| 5-12 years | 16 | (3.3) | **2.20** | **(1.33, 3.62)** |  | 13 | (3.3) | **2.06** | **(1.19, 3.59)** |  | 3 | (1.4) | 2.95 | (0.94, 9.25)^†^ |
| 13-19 years | 31 | (5.6) | 1.13 | (0.79, 1.63) |  | 22 | (5.6) | 0.95 | (0.62, 1.46) |  | 9 | (4.1) | **2.23** | **(1.14, 4.36)** |
| 20+ years | 52 | (2.8) | 0.97 | (0.72, 1.30) |  | 11 | (2.8) | 0.75 | (0.41, 1.37) |  | 41 | (18.9) | 1.08 | (0.76, 1.52) |

aIRR: adjusted Incidence Rate Ratio; FEP: first episode psychosis; 95% CI: 95% confidence interval

^1^Excluding people of foreign-born white British background (n=3) from analyses. Analyses adjusted for sex and interaction with age group. LRT χ^2^ p-value on 4*df*: 6.0, p=0.20 for interaction between age (16-24 vs. 25-35) and age-at-migration.

**Bold** denotes p≤0.05

†p=0.06
